# Intimate Partner Homicide: Comparison Between Homicide and Homicide-Suicide in Portugal

**DOI:** 10.1177/08862605231198007

**Published:** 2023-10-02

**Authors:** Mariana Gonçalves, Eduardo Gomes, Marlene Matos

**Affiliations:** 1 University of Minho, Braga, Portugal

**Keywords:** intimate partner homicide, intimate partner homicide-suicide, intimate partner violence

## Abstract

Intimate partner homicide (IPH) is a tragic event. Studies involving the comparison between IPH and intimate partner homicide-suicide (IPH-S) are scarce, with few studies in Portugal about this issue. The current study aims to compare IPH and IPH-S perpetrators, the victim–perpetrator relationships dynamics, and homicide circumstances. The data was collected through the analysis of 78 judicial processes of IPH that occurred in Portugal, between 2010 and 2015. Of the cases, 51 were IPH, 20 were IPH-S cases, and seven were attempted suicide cases, being perpetrated in 84.6% (*n* = 66) for male perpetrators. Suicide after intimate homicide were all committed by men. All judicial processes analyzed refer to heterosexual relationships. Bivariate and multivariate analyses were performed to compare the groups concerning perpetrator and victim sociodemographic characteristics, victim-perpetrator dyadic dynamics, and crime circumstances. The results show mostly common trends between the two groups with some differentiating factors when compared individually (e.g., perpetrator professional status, criminal records). Regression logistic analysis showed no differences between IPH and IPH-S.

## Introduction

Domestic violence (DV) is a prevalent crime worldwide, a serious public health and social problem, and a serious violation of human rights (Harvey et al., 2007). In the broad spectrum of DV, Intimate Partner Violence (IPV) crimes are characterized as any behavior within an intimate relationship that causes any physical, psychological, or sexual harm, including acts of physical violence, sexual violence, emotional/psychological abuse, and controlling behaviors (World Health Organization, 2012).

Globally, more than one-fifth of women have experienced physical and/or sexual violence from a partner and/or former partner (FRA—European Union Agency for Fundamental Rights, 2014). The most recurrent forms of violence are being pushed, pulling hair, grabbing, or slapping, and beating with a hand or blunt object; this happens for about half of women who are IPV victims and about two-thirds of female victims of former partners (FRA—European Union Agency for Fundamental Rights, 2014). Data on male victims are scarce; statistical reports from the United States and Canada revealed that 22% of men experience some form of intimate violence during their life (e.g., physical, sexual, or psychological), and men from different ethnicity groups than their partners are at higher risk of being victims of IPV (Delta Opposes Violence Everywhere, 2013). Regarding the Portuguese reality, about 28.9% of crimes against people are committed in an intimate relationship context (74.9% female victims and 81% male perpetrators; Internal Security System [Sistema de Segurança Interna], 2021).

IPV ranges from “minor” offenses (e.g., pushes and slaps) to the most extreme outcomes that can result in violent confrontation (e.g., murder; Caman, 2017). Intimate Partner Homicide (IPH) represents the more extreme result of IPV (Matias et al., 2020; Spencer & Stith, 2020) and is defined as the homicide of a person by a spouse or former spouse (Oram et al., 2013). Femicide is the homicide of a woman in an intimate relationship context (Campbell et al., 2003; McFarlane et al., 2002; Sharps et al., 2001). In intimate relationships, the presence of some factors seems to increase the likelihood of a fatal outcome: for example, the presence or facilitated access to firearms by the perpetrator seems to increase eleven times the likelihood of a homicide (Spencer & Stith, 2020). In addition to physical violence, studies also report a high prevalence of psychological violence, controlling, and stalking behaviors (Sharps et al., 2001). Others include violence during pregnancy (McFarlane et al., 2002; Koziol-McLain et al., 2006; Spencer & Stith, 2020) and controlling behaviors (Campbell et al., 2003; Glass et al., 2008) as risk factors for IPV and IPH. Campbell et al. (2003) suggested that controlling behaviors allied to a couple’s recent breakup appeared to increase the risk of femicide by nine times. A meta-analysis that assessed the risk factors to IPH concluded that some risk factors increase the likelihood of lethality in a violent relationship, mostly those related to previous violent dynamics between the dyad victim-perpetrator (e.g., death threats, any kind of threat, stalking; Matias et al., 2020).

In 2019, the United Nations Office on Drugs and Crime (UNODC) conducted a global study on homicide that estimated that 3 out of every 10 women (34%) who are intentionally killed are murdered by their intimate partners. In the family or intimate context, women are at a considerably higher risk of homicide compared to men (UNODC, 2019). In Portugal, a statistical analysis of IPH convictions concluded that between 2007 and 2013, 13% of all homicides were IPH. In addition, there was an increase in the number of female perpetrators (4.7% in 2007 and 17.2% in 2013) and a reduction in the conviction of male perpetrators (93.5% in 2007 and 82.8% in 2013). According to the national annual report of the Internal Security System [Sistema de Segurança Interna] (2021), 15% of the consummate voluntary homicides were committed by an intimate partner.

Literature about homicide topics has historically failed to differentiate between homicide in general and IPH. Despite this, some studies recognized the importance of IPH studies as a distinct typology (Ioannou & Hammond, 2015; Matias et al., 2020). Dobash et al. (2004) argued that IPH perpetrators are more conventional than other violent crime perpetrators. IPH perpetrators display patterns consistent with the general population in childhood victimization, physical violence, psychological violence, as well as educational level and employment, and should, therefore, be treated as a separate subgroup (Dobash et al., 2004, 2009; Weizmann-Henelius et al., 2012). As such, it is possible to adopt the idea that the individual characteristics and risk factors found in general homicide will not be relevant to IPH. However, not all researchers argue that this group of homicides differs from others; for example, Felson and Lane (2010) argued that these are typical violent offenders, not different in their characteristics, experiences, and motivations from those who commit violent crimes outside the family. A study by Smucker et al. (2018), based on a retrospective analysis of court cases, found mortal victims other than the partner in 51 of the 816 cases analyzed (35.2% included the death of a common child, 21.5% of the current partner of the victim, 19.6% friends or housemates, and 9.7% the victim’s parents).

Another IPH typology with significant prevalence is Intimate Partner Homicide-Suicide (IPH-S), where a perpetrator commits suicide after the IPH (Dobash & Dobash, 2015); this phenomenon represents a severe form of interpersonal violence that occurs between couples and families. According to Buteau et al. (1993), IPH-S perpetrators can be divided into two broad groups: generally older individuals with physical and/or economic difficulties, with the primary intention of double-suicide or compassionate homicide, or younger people motivated by jealousy, with a high prevalence of depression and long-term relationships with the victims. In the last group is also more prevalent a history of violent relationships, episodes of separation, personality disorders, and alcohol abuse. More recently, Salari and Sillito (2016) proposed that IPH-S perpetrators can be divided into three categories: young (18–44 years old), middle-aged (45–59 years old), and elder adult (60+ years old); according to the authors, when analyzing the primary intentions, young adults reflect more homicidal motive, with previous IPV history, whereas the elder adults reveal a more suicidal motive, with overwhelming circumstances such as financial stress, depressive symptoms, and others; the middle-aged adults reflect a mix of the other two categories, with most similarities to the young category. In cases of IPH, the perpetrators’ likelihood of committing suicide differs from men to women, with most cases revealing a female victim and a male perpetrator (Bossarte et al., 2006; Holland et al., 2018; Vatnar et al., 2019; Zeppegno et al., 2019).

There are some differentiating characteristics in IPH-S cases when compared to IPH: a higher average age among perpetrators and victims, and the presence of a more formal relationship (spouse or former spouse; Banks et al., 2008; Dawson, 2005; Mathews, 2008); use of alcohol and psychotropic substances by the victim and perpetrator appears to be lower (Banks et al., 2008; Liem et al., 2009; Mathews, 2008); the presence of a firearm seems to be a determining factor (about half of the perpetrators who use firearms in IPH commits suicide, when using another type of weapon the suicide percentage drops to 7%; Smucker et al., 2018). Banks et al. (2008) in a comparative study of homicides and homicides-suicides found that in a representative part of their sample existed a firearm purchase several days before the homicide, suggesting some premeditation. In the absence of firearms, suicide is less common, and homicide is less premeditated with IPH perpetrators using other methods (e.g., asphyxiation, poisoning, incineration, and stabbing).

Compare IPH and IPH-S appears to be challenging; however, some fundamental differences seem to emerge: IPH-S perpetrators appear to be less likely to have a previous criminal record or history of disregard and violations of the law (Vatnar et al., 2019), using much less violent and more premeditated methods (Jensen et al., 2009). Other studies concluded that IPH-S perpetrators have a higher prevalence of mental illness histories (e.g., depression; Cheng & Jaffe, 2019; Jensen et al., 2009; Zeppegno et al., 2019) and have used mental health services (Campbell et al., 2007). Regarding the sociodemographic characteristics, IPH-S perpetrators seem to be mainly native-born citizens and more educated (Vatnar et al., 2019). Also, analyzing the chronology of events, investigations are not clear; some studies apply the term homicide-suicide only to cases where the suicide occurred within 24 hours of homicide, whereas others expand this period to one week (Barraclough & Harris, 2002).

IPH-S in Portugal remains mostly unstudied. Pereira et al. (2010) developed a documentary analysis study based on forensic reports between 2004 and 2008. The analysis revealed 57 cases of dead women, with 26 (46%) being killed by their partners; of this 54% cases (*n* = 14) were IPH-S cases. Regarding the individual characteristics and relational dynamics, this study showed some agreement with previous studies: perpetrators were generally older than the victims, firearms were the most used method, and substance consumption was a prevalent risk factor in IPH and IPH-S cases.

## The Present Study

This study aims to increase current knowledge about IPH and specifically in IPH-S cases, answering questions about individual, situational, and relational characteristics differences between these two groups. So, in addition to filling a gap in Portuguese research on this subject, analyzing these differences may inform and facilitate police forces, and criminal investigators, and help professionals practice. From a practical point of view, the information gathered will contribute to the development of more accurate risk assessment instruments for early identification and the implementation of interventions for potential perpetrators and victims.

The main objective of this study is to perform a comparative analysis of the demographic, situational, individual, and dyadic characteristics that differentiate and define IPH and IPH-S cases. For this, through the documental analysis of judicial processes, we intend to establish comparisons between these two forms of homicide by type of homicide (IPH and IPH-S), sociodemographic variables of the perpetrators and the victims (e.g., age, ethnicity, nationality), the criminal dynamics (e.g., type of weapon used, presence of accomplices or motivation) and the relational history (e.g., prior violence, number of children in common, duration of relationship).

## Methodology

### Sample

For this study, we used a cross-sectional descriptive method. The data was collected based on documentary analysis of 78 judicial processes of IPH, allowing the analysis of an extensive number of variables to determine the predominant characteristics of our sample of IPH and IPH-S offenders, and its validity and quality were demonstrated in a variety of investigative psychology studies (Canter & Alison, 2003).

The data used in this study were obtained through an analysis of IPH judicial cases in Portugal that were finalized, up to date and handed down and/or sentenced between 2010 and 2015. The judicial processes had information about all the judicial phases, namely the police reports, interview transcripts, photographs from the scene of crime, legal and forensic reports, and court transcripts and decisions. Of all the cases, 51 were IPH cases, 20 were IPH-S cases, and 7 were attempted suicide cases, with 84.6% (*n* = 66) male perpetrators. All criminal processes refer to heterosexual relationships.

### Procedure

This study was approved by the Ethics Committee of the University of Minho. In Portugal, the Judiciary Police is responsible for the investigation of all homicides at the national level, so we asked this organization for the identification of all IPH cases that occurred in Portugal in the 5-year period from 2010 to 2015, being identified 150 judicial cases. Of those, 120 were IPH perpetrated by men, and 30 were perpetrated by women. Only IPH offenders aged 18 or over, which corresponds to the age of adulthood in Portugal, were included. The type of victim–offender relationship had not been defined by the team, but we only had access to heterosexual relationships.

After the identification of the judicial processes, the authorizations required to proceed with the project were obtained from the Continental and Islands courts and the judges responsible for each case. Some courts responded immediately willing to collaborate; however, many others did not reply to the authors, despite the continuous insistence. We add access to 60% (*n* = 78) of the total cases identified (*n* = 150), distributed from different regions of Portugal, mainly in urban areas.

The data collection was scheduled and performed in the courts, with an estimated consultation time of approximately 3 hours per process (a total average of 225 hours of consultation). The processes were analyzed through a data collection grid, based on a literature review that was pilot-tested and adapted. This grid is divided into seven main categories: (a) process, criminal procedure, and judicial sentence (e.g., date of occurrence, sentence, conviction); (b) offender (e.g., sex, age, nationality, ethnic group, criminal history, professional status, access/possession of firearms); (c) victim (e.g., sex, age, nationality, ethnic group, professional status, perceived danger); (d) pre-homicidal relational dynamics (e.g., duration of the relationship, prior separation, history of IPV); (e) types of violence and violent behavior (e.g., physical violence, stalking, evidence of frequency); (f) context of homicide (e.g., crime scene, the weapon used, motivation); and (g) expertise (psychological expertise report, psychiatric expertise report, offender social report. The data collected were later converted into 126 quantitative variables, mainly numerical and nominal, that were then entered into database a from the Statistical Package for Social Sciences (SPSS, version 25, IBM SPSS Statistics for Windows (Version 27.0) [Computer software]. IBM Corporation).

### Data Analyses

Descriptive statistics were run to illustrate the characteristics of the sample (see Table 1). A statistical comparison was then run for IPH and IPH-S cases. IPH-S and suicide attempted cases were combined into a single IPH-S group for the analyses. *T-tests* for independent samples were used for numerical variables and *Chi-square* tests for nominal variables. When possible, *Fisher’s* exact test was used to improve the statistical robustness of the analyses. In addition, effect sizes were calculated: *Cohen’s d* for numerical variables (*d)*, *Phi* (ϕ), and *Cramers’ V* for nominal variables.

**Table 1.** Perpetrator’s Characterization: Intimate Partner Homicide (IPH) Versus Intimate Partner Homicide-Suicide (IPH-S).

Perpetrador Characterization	IPH (*n* = 51)		IPH-S (*n* = 27)		*t*	*d*
	*M*	*DP*	*M*	*DP*		
Age	43.98	14.48	56.56	16.12	−3.51**	0.82
	*n*	%	*n*	%	*χ* ^2^	ϕ or *Cramers’ V*
Sex						
Female	12	23.5			7.51	.31
Male	39	76.5	27	100		
Nationality						
Portuguese	38	74.5	22	81.5	1.02	.12
Foreign	13	25.5	4	18.5		
Ethnicity						
Caucasian	37	75.5	25	100	7.31**	.31
Other	12	24.5				
Missing data	2	—	2	—		
Profession						
Undifferentiated	40	90.9	9	64.3	5.74*	.32
Differentiated	4	9.1	5	35.7		
Missing data	7	—	14	—		
Professional status						
Active	20	40.4	6	27.3	12.92*	.43
Unemployed	22	44.9	4	18.2		
Retired	7	14.2	12	54.4		
Missing data	2	—	5	—		
Education level						
Basic education or lower	45	95.7	4	80	2.40	.22
High education or higher	2	4.3	1	20		
Missing data	4		22			
Criminal record						
No	27	54	21	87.5	7.99**	.33
Yes	23	46	3	12.5		
Missing data	1	—	3	—		
Crimes against people						
No	26	64.7	20	83.3	5.03*	.27
Yes	20	35.3	4	16.7		
Missing data	5	—	3	—		
Firearms ownership						
No	33	42.3	13	48.1	2.00	.60
Yes	18	35.9	14	51.9		
Substance abuse						
No	29	56.9	21	80.8	4.32*	.24
Yes	22	43.1	5	19.2		
Missing data	—	—	1	—		
Psychiatric history						
No	43	84.3	20	74.1	1.19	.12
Yes	8	15.7	7	37.9		

* *p* < .05.

** *p* < .01.

Logistic regression models were run, using as dependent variable IPH or IPH-S, to understand the significant differences between the groups and if any of the variables that resulted in significant differences between the groups in the analysis are still significant when taking into consideration the other independent measures. Once the sample size is small, we performed three logistic models to aggregate the variables with significant differences resulting in the chi-square comparisons between IPH and IPH-S regarding the characterization of the perpetrators, the characterization of victims and victimization dynamics, and homicide characterization.

## Results

Table 1 provides a detailed characterization of the comparison between IPH and IPH-S perpetrators. Regarding age, statistically significant differences were found, *t* (76) = −3.51, *p* = .001, *d* = 0.82, with a high effect size; perpetrators in the IPH-S group were older (*M* = 56.56, *SD* = 16.12) than those in the IPH group (*M* = 43.98, *SD* = 14.48). IPH perpetrators were mostly Caucasian (75.5%); however, in the group IPH-S all perpetrators were Caucasian, with a significant difference between the two groups, χ^2^(1) = 7.31, *p* = .006, ϕ = .32, with a small effect size.

IPH and IPH-S groups also differed in the profession, χ^2^(1) = 5.74, *p* = .030, *Cramers’ V* = 0.32, and their occupational status at the time of the crime, χ^2^(2) = 12.92, *p* = .002, *Cramers’ V* = .43. Both groups had higher percentages of undifferentiated occupations; however, the percentage was 90.9% in the IPH group and 64.3% in the IPH-S group. Most IPH perpetrators were unemployed (44.9%), whereas in the IPH-S group, most perpetrators were retired (54.4%). In the IPH-S group, only 6% of perpetrators had a criminal record comparatively to 46% in the IPH group; this difference between the groups was significant, χ^2^(2) = 7.99, *p* = .005, ϕ = .33. Differences were also found between the groups about committing crimes against people, χ^2^(1) = 5.03, *p* = .03, ϕ = .27; 35.3% of IPH perpetrators had been convicted for this type of crimes, compared to 16.7% in IPH-S group. The groups also differed in substance abuse, χ^2^(1) = 4.32, *p* = .05, ϕ = .24; 43.1% of IPH perpetrators experienced substance abuse comparatively to 19.23% of IPH-S perpetrators.

### Victims’ Characterization

Table 2 provides a detailed characterization of the comparison between IPH and IPH-S victims. Statistically significant differences were found in the age, *t*(76) = −2.30, *p* = .02, *d* = −0.54, with a medium effect size; higher mean age was found in the IPH-S group (*M* = 50.56, *SD* = 17.61) compared to the IPH group (*M* = 41.84, *SD* = 14.99). Despite a higher prevalence of female victims in both groups, differences were found between them, χ^2^(1) = 7.51, *p* = .006, ϕ = .31; all IPH-S victims were female compared to IPH victims (76.47%). Differences were also found between groups regarding ethnicity, χ^2^(1) = 6.31, *p* = .013, ϕ = .29. The IPH-S group was composed only of Caucasian victims, which differed from the IPH group (Caucasian victims = 78.43%; other ethnicities = 21.57%). Lastly, the groups statistically differed concerning substance abuse, χ^2^(1) = 5.69, *p* = .03, ϕ = .27; only 3.7% of IPH-S victims showed substance consumption, and this number increased to 24% in the IPH group.

**Table 2.** Victims’ Characterization: Intimate Partner Homicide (IPH) Versus Intimate Partner Homicide-Suicide (IPH-S).

Victim Characterization	IPH (*n* = 51)		IPH-S (*n* = 27)		*t*	*d*
	*M*	*DP*	*M*	*DP*		
Age	41.84	14.99	50.56	17.61	−2.30*	−0.54
	*n*	%	*n*	%	χ^2^	ϕ or *Cramers’ V*
Sex						
Female	39	76.5	27	100	7.51**	.31
Male	12	23.5	.	.		
Nationality						
Portuguese	37	74	23	88.5	2.15	.17
Foreign	13	26	3	11.5		
Missing data	1	—	1	.		
Ethnicity						
Caucasian	40	78.4	25	100	6.31*	.29
Other	11	21.6	.	.		
Missing data	—	—	2	.		
Profession						
Undifferentiated	39	84.8	11	83.3	.00	.002
Differentiated	7	15.2	2	16.7		
Missing data	5	—	14	—		
Professional status						
Active	32	64	10	55.6	.41	.08
Unemployed	11	22	5	27.8		
Retired	7	14	3	16.7		
Missing data	1	—	9	—		
Education level						
Basic education or lower	45	95.7	4	80	.47	.15
High education or higher	2	4.3	1	20		
Missing data	4	—	22	—		
Risk perception						
No	16	50	6	42.9	.20	.06
Yes	16	50	8	57.1		
Missing data	19	—	13	—		
Substance abuse						
No	38	76	26	96.3	5.7*	.27
Yes	12	24	1	3.7		
Missing data	1	—	—	—		
Psychiatric history						
No	42	85.7	18	69.2	2.89	.20
Yes	7	14.3	6	30.8		
Missing data	2	—	3	—		

* *p* < .05.

** *p* < .01.

### Dyad and Violence Dynamics Characterization

Table 3 provides a detailed characterization of the comparison between IPH and IPH-S dyads. Statistically significant differences were found regarding physical violence between the two groups, χ^2^(1) = 8.318, *p* = .006, ϕ = .33. The IPH group presented physical violence in 54.9% of the cases, a higher percentage than the 20% presented by the IPH-S group. Psychological violence, χ^2^(1) = 7.86, *p* = .009, ϕ = .32, and controlling behaviors, χ^2^(1) = 6.61, *p* = .02, ϕ = 30, followed the same pattern, with higher percentages in the IPH group (76.5% vs. 44% and 41.2% vs. 12%, respectively). A marginally significant result was also found regarding stalking behaviors, χ^2^(1) = 3.34, *p* = .02, ϕ = .21, with the IPH group showing a higher percentage (41.2%) of this type of violence when compared to the IPH-S group (20%).

**Table 3.** Dyad Characterization: Intimate Partner Homicide (IPH) Versus Intimate Partner Homicide-Suicide (IPH-S).

Dyad Characterization	IPH (*n* = 51)		IPH-S (*n* = 27)		*t*	*d*
	*M*	*DP*	*M*	*DP*		
Age difference perpetrator–victim	6.26	7.62	6.94	6.08	−0.40	0.04
	*n*	%	*n*	%	χ^2^	ϕ or *Cramers’ V*
Duration of the relationship	12.69	13.28	15.78	12.07	−0.76	−0.24
Number of children in common	0.94	1.03	1.10	1.17	−0.57	−0.25
Type of relationship						
Current	34	66.7	7	25.9	.46	.08
Former	17	33.3	20	74.1		
Previous separations						
No	21	45.7	6	66.7	1.33	.16
Yes	25	54.3	3	33.3		
Missing data	5	—	18	—		
Evidence of a History of IPV						
No	15	30	9	47.4	1.83	.16
Yes	35	70	10	54.6		
Missing data	1	—	8	—		
Primary aggressor						
Perpetrator	26	63.4	10	76.9	.85	.13
Victim	6	14.6	1	7.7		
Both	9	22	2	15.4		
Missing data	10	—	14	—		
Post-separation IPV history						
No	36	85.7	6	54.5	4.94	.31
Yes	6	14.3	5	45.5		
Missing data	9	—	16	—		
Physical violence						
No	23	45.1	20	80	8.32**	.33
Yes	28	54.9	5	20		
Missing data	—	—	2	—		
Psychological violence						
No	12	23.5	14	56	7.86	.32
Yes	39	76.5	11	44		
Missing data	—	—	2	—		
Stalking						
No	30	58.8	20	80	3.34^+^	.21
Yes	21	41.2	5	20		
Missing data	—	—	2	—		
Controlling behaviors						
No	30	58.8	22	88	6.61**	.30
Yes	21	41.2	3	12		
Missing data	—	—	2	—		

*Note.* IPV = Intimate Partner Violence.

+ *p* < .1.

** *p* < .01.

### Homicide Characterization

Table 4 provides a detailed characterization of the comparison between IPH and IPH-S homicide. IPH and IPH-S groups differed regarding the type of weapon used, χ^2^(5) = 16.74, *p* = .005, *Cramers’ V* = .47. IPH perpetrators used preferentially white/cold weapons (56.5%), whereas those who committed IPH-S used mostly firearms (61.5%). A marginally significant result was found in perpetrators’ substance use during the crime, χ^2^(5) = 10.88, *p* = .05, ϕ = .72; 64.3% of the IPH group had alcohol in their system the moment the crime happened, differing from the IPH-S group in which they had mostly psychotropic substances (57.2%). Regarding motivation, 32.6% of the IPH group committed the crime as a result of a fight and the IPH-S group did after infidelity suspicions (30%), the victims’ desire for separation (30%), and victims’ health problems (30%), revealing statistically significant differences between the groups, χ^2^(8) = 22.69, *p* = .004, *Cramers’ V* = .57. A complementary analysis also revealed that in none of the cases in which the primary motivation was the victims’ disease existed prior violence in the relationship. Finally, through court decisions analysis, the groups presented discrepancies regarding the premeditation of the crime; a higher premeditation percentage was observed in the IPH-S group (68.2%) comparatively to the IPH group (40.8%), leading to a statistically significant difference between these groups χ^2^(1) = 4.55, *p* = .04, ϕ = .25.

**Table 4.** Homicide Characterization: Intimate Partner Homicide (IPH) Versus Intimate Partner Homicide-Suicide (IPH-S).

Homicide Characterization	IPH (*n* = 51)		IPH-S (*n* = 27)		χ^2^	ϕ or *Cramers’ V*
	*n*	%	*n*	%		
Previous homicide attempts						
No	47	95.9	20	95.2	.02	.02
Yes	2	4.1	1	4.8		
Missing data	2	.	6	—		
Weapon used						
White/cold	26	56.5	5	19.2	16.74**	.46
Firearm	15	32.6	16	61.5		
House object	3	6.5	.	.		
Physical force	2	4.4	5	19.2		
Missing data	5	—	1	—		
Type of substance						
Alcohol	9	64.3	3	42.9	10.88^+^	.72
Illicit substances	5	35.7	.	.		
Psychotropics	.	.	4	57.1		
Motivation						
Infidelity suspicion	12	24.5	6	30	22.69**	.57
Separation desire (victim)	15	30.4	6	30		
Health problems (victim)	.	.	6	30		
Fear (victim)	1	2	.	.		
Following a fight	16	32.7	2	10		
Debts	2	4.1	.	.		
Health problems (perpetrator)	3	6.1	.	.		
Missing data	1	—	7	—		
Premeditation						
No	29	59.2	7	31.8	4.55*	.25
Yes	20	40.8	15	68.2		
Missing data	2	—	5	—		
Attempt to hide/destroy proofs and/or body						
No	41	80.4	22	91.7	1.54	.14
Yes	10	19.6	2	8.3		
Missing data	—	—	3	—		

+ *p* < .1.

* *p* < .05.

** *p* < .01.

*p* < .001.

### Variables Associated with IPH Versus IPH-S

Regression logistic analysis was performed with variables that significantly differentiate IPH and IPH-S in chi-square analysis. Once the sample size was small, we performed three different logistic regression analyses (Table 5). From the three models tested, only the model that analyzed the predictive value of the independent variables related to victim characteristics and violent dynamics was significant, χ^2^(5) = 18.006, *p* = .003, *R*^2^ Nagelkerke = .29. However, none of the variables were independent predictors of IPH or IPH-S.

**Table 5.** Logistic Regression to Analyze Variables Associated with Intimate Partner Homicide (IPH) Versus Intimate Partner Homicide-Suicide (IPH-S).

Variables associated with IPH vs IPH-S	B	S.E.	Wald	Sig.	Exp(B)	95% C.I. for EXP(B)		χ^2^ (*R*^2^ Nagealkerke)
						LL	UL	
Perpetrators characterization								
Ethnicity	−0.90	1.18	0.58	0.45	0.41	0.04	4.11	χ^2^(6) = 5.87, *p* = .438 (*R*^2^ Nagelkerke = .16)
Profession	1.27	0.86	2.19	0.14	3.56	0.66	19.18	
Professional status	−0.20	0.73	0.07	0.79	0.82	0.20	3.46	
Criminal record	−1.04	1.07	0.95	0.33	0.35	0.04	2.88	
Crimes against people	0.26	0.97	0.07	0.79	1.30	0.19	8.77	
Substance abuse	−0.26	0.90	0.08	0.77	0.77	0.13	4.49	
Constant	−0.09	2.48	0.00	0.97	0.92			
Victim characterization and violent dynamics								
Ethnicity	−1.03	0.87	1.39	0.24	0.36	0.07	1.98	χ^2^(5) = 18.006, *p* = .003 (*R*^2^ Nagelkerke = .29)
Substance abuse	−1.89	1.16	2.65	0.10	0.15	0.02	1.47	
Physical violence	−0.73	0.66	1.22	0.27	0.48	0.13	1.76	
Stalking	−0.86	0.67	1.64	0.20	0.42	0.11	1.58	
Controlling behaviors	−0.95	0.77	1.51	0.22	0.39	0.09	1.76	
Constant	5.87	1.92	9.31	0.00	353.81			
Homicide characterization								
White weapon	1.21	0.81	2.23	0.14	3.34	0.69	16.24	χ^2^(5) = 11.42, *p* = .044 (*R*^2^ Nagelkerke = .21)
Firearm	−0.19	0.76	0.06	0.80	0.83	0.19	3.68	
Separation desire	0.73	0.75	0.95	0.33	2.07	0.48	8.96	
Infidelity suspicion	0.40	0.72	0.30	0.59	1.48	0.36	6.13	
Crime premeditation	1.08	0.69	2.45	0.12	2.95	0.76	11.42	
Constant	−6.11	3.55	2.97	0.09	0.00			

## Discussion

IPH and IPH-S crimes have been extensively studied in the literature; however, they are not usually compared in the same study, with no recent studies comparing these issues in Portugal. There is a consensus among researchers about the heterogeneity of both perpetrators and the crime itself, but few studies have sought to understand the characteristics or combination of features that bring IPH and IPH-S crimes closer or further away. This study sought to analyze the differences and similarities between the crimes of IPH and IPH-S in Portugal between 2010 and 2015.

At an individual level, perpetrators appear to differ from each other in more aspects than victims. IPH-S victims are significantly older than IPH victims, something that has already been demonstrated by Banks and Colleagues (2008). Contrary to what is shown in other studies (Mathews, 2008; Liem et al., 2009), the differences found in substance abuse demonstrate a higher prevalence in the IPH group. The ethnicity analysis also showed differences between the groups: a higher prevalence of Caucasian victims and perpetrators in the IPH-S group is corroborated by the literature, namely Lund et al. (2001) found similar results in their study, with all perpetrators in the IPH-S group being Caucasian. However, most victims in both groups were Caucasian, which may be due to the underrepresentation of minority ethnic communities in Portugal. Also, some factors that could be important features to analyze crime dynamics, such as cultural backgrounds, and other intersectional structural variables are not available in the judicial processes, especially in relation to victims. These limitations in the accessibility and completeness of data limit the knowledge of the social identities of those affected (Cullen et al., 2021), preventing the generalization of data.

Both IPH and IPH-S appear to be gender-stratified phenomena. A higher prevalence of male perpetrators and female victims was found in the IPH group; these results are in line with the results of the annual report of the Internal Security System [Sistema de Segurança Interna] (2021). Concerning the IPH-S group, all perpetrators were male, and all victims were female, revealing differences between the groups; this seems to highlight the gender differences in this type of crime (e.g., Zeppegno et al., 2019), with almost all perpetrators of IPH-S being male. For example, Vatnar et al. (2019) in a study that analyzed 22 years of IPHS, identified only one female perpetrator.

Among perpetrators, individual differences were found in some characteristics. As concluded by Banks et al. (2008), age-related differences were found with a higher average age for IPH-S perpetrators. IPH perpetrators had a higher prevalence of substance abuse than IPH-S perpetrators, and a marginally significant difference was found regarding substance presence during the crime. Previous investigations have found similar facts, Bourget et al. (2000) reported a higher proportion of substance abuse in the IPH group, before and during the crime. Most IPH perpetrators were unemployed. Also, Campbell et al. (2003) reported that occupational status was one of the main risk factors for IPH; on the other hand, most IPH-S perpetrators were retired at the time of the crime, which may be justified by their higher average age. The fact that IPH perpetrators have a mostly undifferentiated profession or are unemployed, seems to indicate that socioeconomic status is a strong predictor of IPH but not IPH-S. Results from a mini review about homicide-suicide (Zeppegno et al., 2019) revealed that only one study associated homicide-suicide with lower levels of economic level, unemployment, and educational level (Reckdenwald & Simone, 2016). Also, Vatnar et al. (2019) found that IPH-S perpetrators are less socially marginalized and more often employed. However, in this study, multivariate analyses showed that, when analyzed together, these factors did not differentiate IPH and IPH-S perpetrators.

The existence of a criminal record is considered a risk factor for IPH (Belfrage & Rying, 2004). However, this is less common in IPH-S cases (Eliason, 2009; Vatnar et al., 2019), which is corroborated by the present study. These results allied with the presence of violence in the relationship (physical and psychological violence and controlling behaviors) seem to indicate that IPH perpetrators are more violent in and out of the relationship than those who commit IPH-S. Despite these results, it was not found differences regarding DV history; both groups reported high levels of previous violence in the relationship context. It is important to note that although there is no evidence of DV in some processes, we cannot affirm that there was no such history in these cases, but also that there was no evidence of that in the processes.

Motivation appears to be a differentiating factor between the groups; according to Jensen et al. (2009), IPH-S perpetrators would use less violent methods, have suicide or double-suicide as their primary intention, and would be mainly motivated by the victims’ terminal or chronic disease. However, with a lower number of cases, in this study we found some cases of the victim’s illness as being the possible motivation for the crime for IPH-S; however, infidelity suspicion and the victims’ separation desire were also important motivators. However, due to the small amount of information available in criminal processes about this topic, this should be interpreted with caution. Additionally, we found that in none of the cases in which the primary motivation was the victims’ disease, there was evidence of previous violence in the relationship, and that these cases are, probably, even less common.

Regarding the type of weapon used, the results were consistent with previous studies (Belfrage & Rying, 2004; Bourget et al., 2000; Dawson, 2005; Salari & Sillito, 2016; Siems et al., 2017; Zeppegno et al., 2019), that reported a higher presence of firearms in IPH-S cases. Premeditation appears in the literature as a preponderant feature of IPH-S perpetrators; Dawson (2005) verified that existed premeditation in more than 50% of IPH-S cases, whereas in IPH only existed in 20% of the cases. In this study, it was possible to observe this difference, however in a less robust way.

Despite these results, no significant results were found in some characteristics considered by the literature as differentiators between the groups: suicide as primary intention; a higher proportion of formal relationship in the IPH-S group; more depression in the IPH-S group; and a bigger age difference between perpetrator and victim in IPH-S group (Banks et al., 2008; Liem et al., 2009; Jensen et al., 2009; Mathews, 2008). The fact that this study was unable to verify these differences may be due to some limitations: the number of processes analyzed, although substantial for the Portuguese reality, may not be representative of the target population; additionally, the little information present in the processes, especially in IPH-S cases, led to a high number of missings in some of the variables (e.g., evidence of mental health problems, evidence of DV, previous contact with police and social services, criminal records), removing statistical robustness from the analyses. In fact, IPH-S judicial processes have little information due to the crime outcome. Although a criminal proceeding is instituted, it is extinguished by the death of the perpetrator. In this way, the scarcity of information is because there is no need to collect evidence within the scope of the judicial process to judge the perpetrator. Also, a better organization and systematization of the official information present in the processes would be important for a better and more effective analysis. However, despite these limitations, the results gathered allowed the visualization of the problem from a new point of view, informing about possible interventions, and new prevention strategies. Also, since this type of crime appears to be less linked to an escalation of prior violence, future investigations about IPH-S perpetrators’ motivations and how these may be linked to mental disorders would be important.

## Conclusions and Practical Implications

Given the results obtained, we can conclude that both groups mostly present common patterns between them; however, the distinguishing characteristics may be important from a practical point of view. Including this information in risk assessment tools can increase their effectiveness and help in the early identification of these cases, facilitating the work of police forces and victim support institutions. Both groups present a high prevalence of prior violence in the relationship, demonstrating the continued need for intimate violence prevention programs. Multidisciplinary cooperation also seems to be essential in these cases, especially among justice, health, and social systems. Specialized training and paying more attention to these cases are the key to improving the effectiveness of professional practices.

Relatedly to the research agenda, although there is a concern of the authors to consider diversity in this study, the judicial processes present scarce information about intersectional factors that could better explain the dynamic of the crime, limiting the inclusion of diversity and inclusion in the study of violence as suggested by some authors (e.g., Bent-Goodley, 2021). Also, once the authors did not have access to judicial processes of same-sex couples, future research projects should try to include these intersectionality factors, to better understand the dynamics of this crime. More studies with larger samples must be performed; however, the information on the judicial processes must be complemented with other informants (e.g., professionals, families of the victims and perpetrators in case of IPH-S; victims of IPH temptations and perpetrators).
